# Hepatoprotective Activity of *Ficus carica* Leaf Extract on Rifampicin-Induced Hepatic Damage in Rats

**DOI:** 10.4103/0250-474X.43003

**Published:** 2008

**Authors:** N. Y. Gond, S. S. Khadabadi

**Affiliations:** Department of Pharmacognosy, Nanded Pharmacy College, Nanded-431 605, India; 1 Government College of Pharmacy, Amravati-444 603, India

**Keywords:** *Ficus carica*, hepatoprotectice activity, rifampicin-induced hepatic damage

## Abstract

Shade dried leaves of *Ficus carica* were extracted using petroleum ether (60-80°) and tested for antihepatotoxic activity on rats treated with 50 mg/kg of rifampicin orally. The parameters assessed were serum levels of glutamic oxaloacetate transaminase, glutamic pyruvic transaminase, bilirubin and histological changes in liver. Liver weights and pentobarbitione sleeping time as a functional parameter were also monitored. There was significant reversal of biochemical, histological and functional changes induced by rifampicin treatment in rats by petroleum ether extract treatment, indicating promising hepatoprotective activity.

Liver diseases remain one of the serious health problems. In the absence of reliable liver protective drugs in allopathic medical practices. Herbs play important role in the management of various liver disorders^1^. However, in ayurveda many indigenous plants have been used as hepatoprotective agents. A number of reviews are published stating the importance of plant drugs in the diseases of liver. Indigenous plant *Ficus carica* was selected for investigating hepatoprotective activity^2^,^3^.

*Ficus carica* Linn. (Moraceae), commonly known as *Anjir*. The plant is considered to be a native of carica in Asia Minor and is grown in nearly all tropical and sub-tropical countries. In India its commercial production is limited to a few centers near Pune^4^,^5^. During casual conversation with tribal people of Maharashtra, it was found that they chewed the leaves of *Ficus carica* to treat Jaundice. However, no scientific work has been carried out on the leaves of * Ficus carica* to prove the hepatoprotective activity.

*Ficus carica* leaves were identified by comparing them with Herbarium and by carrying out their microscopical and physical evaluation study. The leaves of *Ficus carica* were collected from Nanded district, Maharashtra. The leaves were dried in the shade, powdered and used for extraction^6^.

The petroleum ether (60-80°) extract of the above crude drug was prepared by Soxhlet extraction. The extract was tested for hepatoprotecive activity in rats. Four groups (1-IV) of six rats each, of either sex weighing between 150-200 g, were selected^7^. Group-I was normal control, fed with vehicle for 60 days. The animals in groups II and IV were orally administered with rifampicin 50 mg/kg for 60 days^8^,^9^. Animals in group III were administered orally the extract of * Ficus carica* leaf (200 mg/100 g) for 10 days. To animals in the group-IV petroleum ether extract of *Ficus carica* was given for after 61 days for 10 days. On the 71^st^ day, pentobarbitione sleeping time was determined after which animals were weighed and sacrified. The liver was examined morphologically, dissected out, weighed and stored in 10% formalin. Blood was collected by cardiac puncture, serum separated out and used for estimation of SGOT, SGPT by the Reitman and Frankel method, bilirubin by the Malloy and Evelyn method^10^. The sleeping time was assessed by injecting pentobarbitione sodium (30 mg/kg, ip) in water for injection^11^. The sleeping time, weight of liver and biochemical parameters are all presented in Table 1. All animal experimental protocols have been approved by the Animal Ethics Committee (CPCSEA Reg. No. is 40/1999/CPCSEA.

**TABLE 1:** EFFECT OF PETROLEUM ETHER EXTRACT OF *FICUS CARICA* LEAF ON RIFAMPICIN-INDUCED HEPATOTOXICITY

Groups	Pentobarbitone sleeping time (min)	Liver weight (g)	Serum bilirubin (mg%)	SGPT (units/l)	SGOT (units/l)
Group-I (control)	140±2.28#	3.08± 0.084#	0.77±0.019#	63±2.800#	181±1.549#
Group-II (rifampicin treated)	195±3.56*	4.86±0.089*	0.94±0.029*	88±1.048*	289±0.894*
Group-III (petroleum ether extract)	147±2.78	3.28±0.057	0.78±0.009	64±2.316	200±3.386
Group-IV (rifampicin+ petroleum ether extract)	143±2.25**	3.18± 0.018**	0.80±0.019**	66±2.440**	184±2.16**

All values are mean±SEM, n=6, P values: P<0.05, when compared to group-II^*^ vs group-IV^**^ and group-II^*^ vs group-I^#^

Histopatholigical examination of the liver sample was carried out by taking thin transverse section with the help of microtome and permanent slides were prepared with Ehrlich’s hematoxylin and eosin stain. The slides were examined under microscope. Histology of normal liver, damaged liver and recovered liver was studied and compared.

Liver is a versatile organ in the body concerned with regulation of internal chemical environment. Therefore, damage to the liver inflicted by hepatotoxic agents is of grave consequence. There is an ever increasing need for an agent which could protect liver from damage, especially of one which facilitates regeneration by proliferation of parenchymal cells after damage arrest growth of fibrous tissue. As such liver is highly affected primarily by toxic agents such as ccl_4_, paracetamol, D-galactosamine, alcohol, rifampicin and thioacetamide through different mechanisms.

In the present study, rifampicin is used as a hepatotoxic agent. Rifampicin is a major drug used for treatment of tuberculosis, but its chronic use is known to cause hepatotoxicity. The mechanism of hepatotoxicity induced by rifampicin is that it competes with bilirubin for transport across the liver cell and conjugated or unconjugated hyperbilirubinaemia can often occur in chronic hepatitis induced by rifampicin.

It was observed from the morphological examination of liver that treatment with rifampicin resulted in liver enlargement, which turned pale brown in colour. The group treated with petroleum ether extract of *Ficus carica* had livers which were similar to that found in the normal rats and were healthy in appearance.

Pentobarbitione sleeping time prolonged in rifampicin treated group. In spite of a large biological variation in sleep time, it was observed that the sleep time was reduced in the treated group. In the group of animals administered with petroleum ether extract the sleeping time was decreased as compared to rifampicin treated group and almost restored back to the initial sleeping time. For the study of liver weight and biochemical parameters, all the values obtained were subjected to statistical analysis. A significant reduction in liver weight was observed in group treated with petroleum ether extract of *Ficus carica*.

The result of the biochemical tests revealed the elevation of serum enzyme level in rifampicin treated group compared to control group, indicating that rifampicin induced liver damage. A significant reduction was observed in SGPT, SGOT levels in the group treated with extract of *Ficus carica*.

The histological study showed recovery of the damaged liver cells in the drug treated group. The ruptured cells of intoxicated liver were reformed. The cytoarchitecure was restored to the same as normal liver. The protective effect appears to be maximum with extract of *Ficus carica*. Hence it is concluded that the petroleum ether extract of *Ficus carica* definitely possess liver protective activity. However, the data obtained in the present study appears to support traditional use of the medicinal plant in the treatment of liver diseases.
